# A Case of Singular Abstinence

**Published:** 1808-11

**Authors:** Robert Taylor

**Affiliations:** Member of the Royal College of Surgeons, London. Lane End, Staffordshire


### *To the Editors of the Medical and Physical Journal.*

GENTLEMEN,

THOUGH I have declined the practice of my profession, I shall ever consider it my duty to promote its interests, and to contribute the mite which Providence may put in my way for the good of society, and the advancement of science. I trust I am solely actuated by this principle, in eliciting, through the medium of your liberal miscellany, the opinions and theories of my medical brethren, on the remarkable facts which I have been engaged to ascertain. There is now living in the village of Tutbury, in Staffordshire, a woman named Ann Moor, in whom nature appears to have established a mere circulating recumbent life, without the usual essential of nutricious juices. It appears from her asseverations, which I am compelled to admit on the subsequent testimony of actual demonstration, that this striking variety of constitution has been the work of many years. The consistency of her whole narration, as to the main fact, is itself a forcible evidence of her integrity. But I have taken pains to give it all the confirmation that a human circumstance could admit, or the most determined incredulity suggest. All the persons formerly about her, have been removed, and she has been taken to the house of a most decided objector to her veracity; and two persons in succession have watched day and night. Placards have been stuck up, maintaining these facts; and the sceptical invited to witness or take part in the investigation.

It has been announced in the Derby paper, and the medical men of that place acquainted with it, both by letter and personal interview. But as to evidence, I need not affirm further, than that it has not left an individual in the place unsatisfied, and remains at this time a notorious fact that that continues to invite the inquiry, and challenges disproof before all the world. She had been declining in health a long time, and thinks she had not been an hour free from pain in her left side for nine years previous to her first attack of Anorexy; which she imputes to her washing out the linen of a person affected with scrofulous ulcers; in consequence of which, she lost all desire of food, and yielded her work on the 4th of November, 1806. From that time till March, the amount of sustenance taken did not exceed the ratio of ℥ß per diem, her strongest drink being tea, but without milk or cream; whatever she took, recalled to her imagination the strong smell of corruption, which at first disordered her; and the slimy matter which she frequently vomited up from the mere recurrence of the idea, seemed to have the appearance and scent that had offended.

In March, 1807, she was afflicted with strong fits, which usually left the cramp in her stomach; to remedy which, she drank boiling hot gruel, which, though it scalded her lips, had no disagreeable effect on her stomach; and any thing of inferior heat gave a sense of cold, and caused rigors. She first took to her bed, for a continuance, on the 14th of April, 1807.

On the 20th of May following, she attempted to swallow a bit of biscuit, which was immediately rejected with dreadful vomiting, and blood.

In the latter end of June, she took the last substance she ever swallowed, being a few black currants. Her last evacuation, (e recto) was by diarrhœa, and took place on the 3d of August. Since which time, she has fallen off also in the quantity of fluids, omitting to take any (at times) two days together. Her common tea has been once varied for onion tea. Her strength she allows to have decreased, but her spirits and mental energy never have, though she is frequently taking cold from the slightest causes. Nor is her head ever free from pain.

In the course of the first three days of the investigation, she swallowed in the whole about ℥iß of water: but happening to step into the room while she was swallowing it, the extreme misery of deglutition, and the violent rising of wind resisting its passage to a degree that almost seemed to threaten suffocation, induced me to dissuade her from taking any more while the experiment that was to vindicate her veracity continued. She has followed my advice, and finds every good effect attained from the occasional cleansing her mouth with a moistened rag; as the former object had been only to relieve her of a sickly faintish taste in the mouth. There has lately been a slight appearance of the menses, which she had thought completely to have ceased. She renders an average of a pint of urine in two days; which is very offensive, and of a high colour; and her skin is always moist. But the greatest phænomenon is her extreme emaciation, though she has less of the facies hipocratica than is common to consumptive patients, and is remarkably cheerful and urbane, possessing a far greater stock of ideas and intelligence than is to be found commonly in her sphere of life. Her circumference, measured rouud the loins, is 20½ inches, across the chest 28½, and across the hips 30 inches. There is scarcely the trace of any viscus to be felt in the abdomen; the bladder, uterus, and its appendages, are sunk beneath the arch of the pubes, and every thing else (that might be) is drawn up under the ribs, so that it cannot be perceived. From the lowest rib, the integuments descending to the ossa illii form an empty cord-like folding, and at the umbilicus the flaccid parietes abdominis may be readily rubbed over the lumbar vertebræ, and no kind of substance felt to intervene.—The grand trunk of the aorta may be traced by the finger from the place most immediately under the ensiform process of the sternum, where the loose integument is drawn down upon it, nearly to its bifurcation. It may be drawn a little from its situation over the spine, and thus by holding the skin across it with my thumb and finger, I have been able to make it apparent to the bye-standers, as they thus saw both its shape and pulsation. The watches have been faithfully kept, and (whatever may have wrought the difference if it exists) she says she thinks she is better and stronger than she has been these six months, and is certainly improved in health since her removal; her pulse has kept the standard of health, with daily exacerbations. She sleeps well, and enjoys a remarkably serene and happy mind. Her voice is strong, and holds out the full female exercise of that faculty. Her muscular power is such that she can conveniently raise, and support herself in bed. Thus, Gentlemen, the watch sitting at the time that I write this, (which must cease to morrow, as I engaged to see the woman safely returned to her habitation before I returned home) it is now the 16th day that she has been under the strictest scrutiny; and the 13th day that she has abstained from all fluids. She is now better in health than when the examination was instituted; and as far as from the corroborating testimony testimony of this evidence her veracity may be admitted, the 14th month that she has subsisted altogether without aliment.

I have simply stated facts, which, in the hands of the exalted Lovers of Physiology that read your Journal, may be in the way of rendering that assistance to Philosophical Research, which will amply remunerate my labour. I would forbear myself offering any theory, being confident of my incompetence, and that even the pursuit of such an object, would rather lessen the validity in the eyes of men of science, of what might have been better established by a fair and unbiassed narration. But in committing this to your care for publication, I shall anxiously wait for instruction from others, in the channel through which it has so often flowed to me.

I am, &c.

ROBERT TAYLOR,
Member of the Royal College of Surgeons, London.

*Lane End, Staffordshire,*
*Sept.* 28, 1803.

